# 13 Juvenile idiopathic arthritis and cardiovascular risk factors

**DOI:** 10.1093/rheumatology/keac496.009

**Published:** 2022-10-07

**Authors:** Houssem Tbini, Safa Rahmouni, Soumaya Boussaid, Ahlem Ben Ammou, Samia Jemmali, Sonia Rekik, Khaoula Zouaoui, Hela Sahli, Mohammed Elleuch

**Affiliations:** 1 Department of Rheumatology, La Rabta Hospital, Tunis, Tunisia; 2 University of Tunis El Manar

## Abstract

**Background:**

Juvenile idiopathic arthritis (JIA) is the most common pediatric rheumatic disease. Although, some patients achieve remission, some cases of JIA may persist into adulthood. Patients with JIA and other inflammatory joint diseases have increased cardiovascular disease risk compared with the general population.

**Objectives:**

To study the cardiovascular risk factors in JIA and their association with disease parameters.

**Methods:**

This was a retrospective study including adults with long-standing JIA according to the International League of Associations for Rheumatology (ILAR) criteria over a period of 28 years (1994–2022). We collected sociodemographic and anthropometric parameters, clinical data, results of biological assessments, and data on prescribed therapies.

We studied the following cardiovascular risk factors: family history of cardiovascular event, physical inactivity, smoking, arterial hypertension, diabetes, dyslipidaemia and obesity.

**Results:**

We included 29 patients. The M/F sex ratio was 0.71, the mean age was 35.69 ± 11.72 [18–61] years. The mean age of disease onset was 11.10 ± 4.25 [2–16] years. The average diagnostic delay was 52.96 ± 95.97 [0–336] months. The average disease duration was 24.48 ± 12.76 [1–47] years. The mean BMI was 21.20 ± 4.88 kg/m2 [14.17–27.55].

The polyarticular form was the most frequent, noted in 55.2% of cases (*n* = 16). Extra-articular manifestations were observed in 55.2% of cases. Mean CRP was 42.74 ± 63.37 [2–218] mg/l and biological inflammatory syndrome was present in 19 cases. Rheumatoid factor, ACPA and anti-nuclear antibodies were observed in 12, 7 and 5 cases respectively.

Corticosteroid therapy and NSAIDs were prescribed to 18 of the subjects.

Cardiovascular risk factors were present in 41.4% (*n* = 12) of cases: family history of cardiovascular event (*n* = 2 cases), physical inactivity (*n* = 5 cases), smoking (*n* = 3 cases), arterial hypertension (*n* = 4 cases), diabetes (*n* = 4 cases), dyslipidaemia (*n* = 4), and BMI ≥ 25 kg/m² (*n* = 4).

Following parameters were significantly higher in patients with cardiovascular risk factors: the presence of a biological inflammatory syndrome (81.8% *vs* 35.3%; *p* = 0.016), the frequency of prescription of corticosteroids (91.7% *vs* 52.9%; *p* = 0.026) and NSAIDs (83.3% *vs* 47.1%; *p* = 0.047).

However, no significant difference was noted when comparing these parameters: gender, age, age of disease onset, disease duration and presence of extra-articular manifestations. Moreover, cardiovascular risk factors were not associated with the presence of rheumatoid factor, ACPA, and antinuclear antibodies.

**Conclusion:**

Inflammation, corticosteroid therapy and NSAIDs are associated with the presence of cardiovascular risk factors in JIA. The evaluation and control of this risk must be regular during patient follow-up. Control of inflammation and rationalization of treatment are necessary.

